# Development and Validation of a Robust Pyroptosis-Related Signature for Predicting Prognosis and Immune Status in Patients with Colon Cancer

**DOI:** 10.1155/2021/5818512

**Published:** 2021-11-18

**Authors:** Zhicheng Zhuang, Huajun Cai, Hexin Lin, Bingjie Guan, Yong Wu, Yiyi Zhang, Xing Liu, Jinfu Zhuang, Guoxian Guan

**Affiliations:** ^1^Department of Colorectal Surgery, The First Affiliated Hospital of Fujian Medical University, Fuzhou, Fujian, China; ^2^Department of Radiation Oncology, Fujian Medical University Union Hospital, Fuzhou, Fujian, China

## Abstract

**Background:**

Pyroptosis has been confirmed as a type of inflammatory programmed cell death in recent years. However, the prognostic role of pyroptosis in colon cancer (CC) remains unclear.

**Methods:**

Dataset TCGA-COAD which came from the TCGA portal was taken as the training cohort. GSE17538 from the GEO database was treated as validation cohorts. Differential expression genes (DEGs) between normal and tumor tissues were confirmed. Patients were classified into two subgroups according to the expression characteristics of pyroptosis-related DEGs. The LASSO regression analysis was used to build the best prognostic signature, and its reliability was validated using Kaplan–Meier, ROC, PCA, and t-SNE analyses. And a nomogram based on the multivariate Cox analysis was developed. The enrichment analysis was performed in the GO and KEGG to investigate the potential mechanism. In addition, we explored the difference in the abundance of infiltrating immune cells and immune microenvironment between high- and low-risk groups. And we also predicted the association of common immune checkpoints with risk scores. Finally, we verified the expression of the pyroptosis-related hub gene at the protein level by immunohistochemistry.

**Results:**

A total of 23 pyroptosis-related DEGs were identified in the TCGA cohort. Patients were classified into two molecular clusters (MC) based on DEGs. Kaplan–Meier survival analysis indicated that patients with MC1 represented significantly poorer OS than patients with MC2. 13 overall survival- (OS-) related DEGs in MCs were used to construct the prognostic signature. Patients in the high-risk group exhibited poorer OS compared to those in the low-risk group. Combined with the clinical features, the risk score was found to be an independent prognostic factor of CC patients. The above results are verified in the external dataset GSE17538. A nomogram was established and showed excellent performance. Gene ontology (GO) and Kyoto Encyclopedia of Genes and Genomes (KEGG) analyses indicated that the varied prognostic performance between high- and low-risk groups may be related to the immune response mediated by local inflammation. Further analysis showed that the high-risk group has stronger immune cell infiltration and lower tumor purity than the low-risk group. Through the correlation between risk score and immune checkpoint expression, T-cell immunoglobulin and mucin domain-containing protein 3 (TIM-3) was predicted as a potential therapeutic target for the high-risk group.

**Conclusion:**

The 13-gene signature was associated with OS, immune cells, tumor purity, and immune checkpoints in CC patients, and it could provide the basis for immunotherapy and predicting prognosis and help clinicians make decisions for individualized treatment.

## 1. Introduction

Colon cancer (CC), a common malignancy arising from the digestive system of mankind, exhibited an obviously rising tendency in both morbidity and mortality [1]. Dietary habits, age, obesity, smoking, and lack of physical exercise are well-known risk factors for colon cancer. The most common subtype of colon cancer is colon adenocarcinoma (COAD) which accounts for 98% of newly diagnosed colon cancer cases with a 5-year survival rate of 40–60% [2]. For therapeutic effect, there are still significant individual differences among patients with CC. the reason is not only associated with socioeconomic factors but also associated with individual genetic heterogeneity [3]. Therefore, it is obviously a great challenge to investigate and develop new strategies for the CC early diagnosis, more precision therapy, and predicting prognosis. Currently, the tumor-node-metastasis (TNM) stage, based on anatomical information, is a common tool to evaluate the prognosis of patients. Nevertheless, the great limitation of the TNM stage is that it may not fully consider the genetic heterogeneity within individual tumors. With the development of sequencing technology, there is a deeper understanding of the transcriptomes of tumors. Assessment of KRAS and BRAF mutation status or MSI status is widely used in clinical treatments. This makes CC patients diagnosed in the middle to the late stage have more treatment opportunities than before [4]. However, because of the complexity of the molecular mechanism affecting the prognosis of CC, single gene/factor prediction models are often accompanied by low accuracy. In contrast, polygene-based models tend to show better results in predicting the prognosis of various cancers [5–7]. Therefore, we need a reliable prognostic gene signature to promote individualized therapy and help survival prediction for CC patients.

Pyroptosis, also known as cell inflammatory necrosis, is a kind of programmed cell death, which is characterized by the continuous expansion of cells until the rupture of the cell membrane, leading to the release of cell contents and activating a strong inflammatory response [8]. Pyroptosis occurs when activated caspase-1 cleaves the protein gasdermin D, releasing the gasdermin N subunit, which can form a pore in the plasma membrane [9]. Pyroptosis is closely related to a variety of diseases; for tumors, it is a double-edged sword. On the one hand, as an innate immune mechanism, pyroptosis can inhibit the development of tumors, and on the other hand, as a proinflammatory cell death mode, pyroptosis, in turn, provides a suitable microenvironment for tumor growth [10]. The long-term chronic inflammatory response can lead to local tissue dysplasia and thus carcinogenesis. Especially, considering that the presence of a large number of bacteria in the intestine may increase the chance of infection with the occurrence of pyroptosis. Therefore, we hypothesized that pyroptosis might play an important role in the development of colon cancer. Although, up to now, several studies have linked pyroptosis with colon cancer [11–13], there are still few scientific and clinical studies on the correlation between CC and pyroptosis; whether pyroptosis is correlated with CC prognosis and identifies expression characteristics of the key pyroptosis-related genes (PDGs) in CC progression remains largely unknown. Despite significant progress in CC gene signatures, few have considered the use of pyroptosis-related gene characteristics to construct a prognostic signature in CC. Accordingly, we carried out a systematic study on pyroptosis-related genes to explore the expression characteristics of those in normal and tumor tissues and predict the prognosis and immune response of patients by trying to construct a prognostic signature.

## 2. Materials and Methods

### 2.1. Acquisition of Data

The level 3 RNA-seq data (Workflow Type: HTSeq-FPKM) of 385 COAD patients and the corresponding clinical information were obtained from The Cancer Genome Atlas (TCGA) dataset (https://portal.gdc.cancer.gov/), in which the method of acquisition and application complied with the guidelines and policies. FPKM values were then normalized by log2 (FPKM + 1) for the subsequent analysis. The GSE17538 gene expression profiles were acquired from the Gene Expression Omnibus (GEO: https://www.ncbi.nlm.nih.gov/gds/) database, including exhaustive transcriptome information about 238 cases of colon cancer patients (Platform: GPL570). The original datasets extracted from GSE17538 were normalized with the RMA method. Both TCGA and GEO databases are publicly available; thus, ethical approval is not required for the present study.

### 2.2. Identification of Differentially Expressed PRGs

A total of 33 pyroptosis-related genes were obtained from previous reports [14]. Among them, GSDMA is deleted because there is no annotation information about it in the GPL570 platform. The “limma” package was used to identify DEGs between tumor and adjacent normal tissues in the TCGA cohort with a *P* value <0.05. Heatmap of DEGs was plotted by “pheatmap” package. PPI networks of DEGs were constructed using STRING v11.5 (http://string-db.org/) with default parameters (confidence = 0.4). Pearson's correlations among DEGs were calculated using “reshape2” package (cutoff = 0.2), and the correlation networks were generated using “igraph” package. The MCODE plug-in in Cytoscape software was used to identify the hub genes of the PPI network.

### 2.3. PRGs-Based Classifications of CC Patients in the TCGA and GSE17538 Cohorts

Unsupervised consensus clustering, an algorithm based on k-means machine learning, was utilized to explore a molecular classification of both the TCGA and GSE17538 CC cohorts based on the expression patterns of PRGs using the “ConsensusClusterPlus” package [15] in R. The optimal number of clusters is determined according to the consensus score and the relative change of the area under the CDF curve of the consensus heatmap. Then, Kaplan–Meier survival analysis was performed to evaluate the prognosis of patients in different MCs. We also performed comparisons of the clinicopathological variables and the difference of tumor immune microenvironment between different clusters of patients to further explore the associations between the PRGs-based MCs and the clinical features or local immune status of CC patients.

### 2.4. Development and Validation of the PRGs-Based Prognostic Risk Signature

We analyzed the differences between patients with different clusters in the TCGA cohort and the GSE17538 cohort to obtain intersecting genes (*P* < 0.05). Then, based on the TCGA cohort, we used univariate Cox regression analysis to screen the genes related to prognosis by setting a strict significance threshold (*P* < 0.0001). Afterward, LASSO Cox regression analysis was performed to construct a prognostic signature with minimizing the risk of overfitting. The risk score of the patients is calculated according to the normalized expression level of each gene and corresponding regression coefficient as the following formula: *Risk score* = ∑ Coefi ∗ Expri. Then, patients were divided into the high-risk group and the low-risk group according to the median risk score. The survival curve was drawn between the high-risk group with the low-risk group by using the “survival” and “survminer” packages of the R software, and the accuracy of the signature is evaluated using the ROC curve. PCA and t-SNE were used for dimensionality reduction analysis to assess the ability to distinguish different risk patients of the risk signature. The stability of the risk signature is verified by the GSE17538 cohort.

### 2.5. Construction and Validation of a Predictive Nomogram

The Cox regression analysis was performed to determine whether the risk score and relevant clinical parameters could be predictors associated with OS for CC patients. Considering the collinearity among the clinical variables, we excluded the T/N/M stage and retained the AJCC stage. Subsequently, based on the results of multivariate Cox regression analysis, a prognostic nomogram was generated to predict 1-year, 2-year, and 3‐year OS of CC patients in the TCGA cohort. The predicted OS of the nomogram against observed survival rates was plotted using the calibration curve.

### 2.6. Functional Enrichment and Immune Characterization Analysis

The “limma” R package was used to identify DEGs between the high-risk and low-risk groups (*P* < 0.05). Gene Ontology (GO) including biological process (BP), cellular component (CC), and molecular function (MF), and Kyoto Encyclopedia of Genes and Genomes (KEGG) pathway enrichment analyses of the DEGs were performed using the “clusterProfiler” R package. In order to compare the immune status between different groups. The “gsva” package was utilized to conduct the ssGSEA to calculate the scores of infiltrating immune cells and to evaluate the activity of immune-related pathways. Estimate, immune, and stromal scores of each patient were calculated with the ESTIMATE algorithm of the “estimate” package [16] to evaluate the difference of immune microenvironment. The correlation between the expression of common immune checkpoints and risk score was analyzed by drawing a correlation matrix diagram.

### 2.7. Statistical Analysis

All statistical analyses were accomplished with R software (v4.0.3). Continuous variables were presented as mean ± standard deviation (SD) as appropriate. Normally and nonnormally distributed variables were analyzed using the unpaired Student's *t*-test and the Wilcoxon test, respectively. A hazard ratio (HR) and a 95% confidence interval (CI) were evaluated by univariable and multivariate Cox regression models. The statistical value *P* < 0.05 indicates that the difference is statistically significant (∗*P* value < 0.05, ∗∗*P* value < 0.01, ∗∗∗*P* value < 0.001).

## 3. Result

### 3.1. Identification of DEGs between Normal and Tumor Tissues

**Figure 1:**
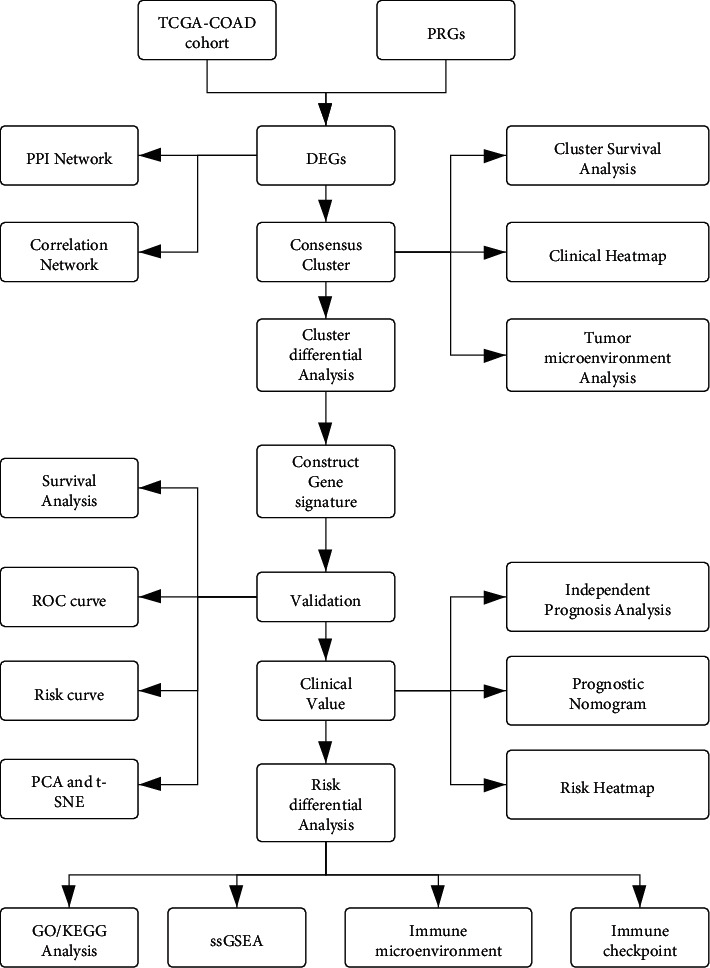
The workflow of the study (PRGs: pyroptosis-related genes, DEGs: differentially expressed genes).

**Figure 2:**
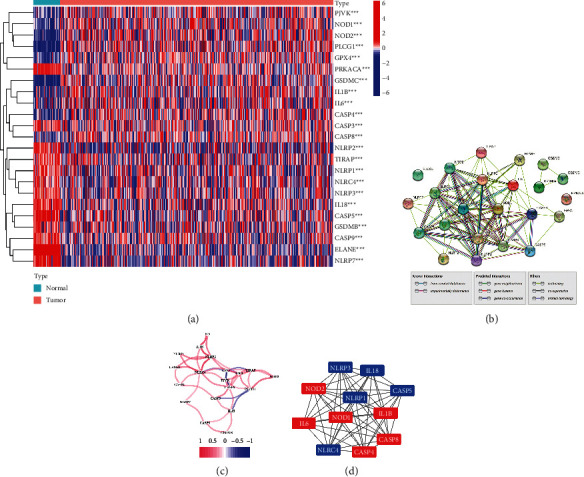
Expression characteristics of 32 pyroptosis-related genes. (a) Heatmap of the pyroptosis-related gene expressions between the normal and the tumor tissues. (b) PPI network exhibiting the interactions of the pyroptosis-related genes (interaction score = 0.4). (c) The correlation network of the pyroptosis-related genes (red line: positive correlation; blue line: negative correlation. The depth of the colors reflects the strength of the relevance). (d) 11 hub genes identified by the MCODE (red: upregulation in tumor; blue: downregulation in the tumor).

**Figure 3:**
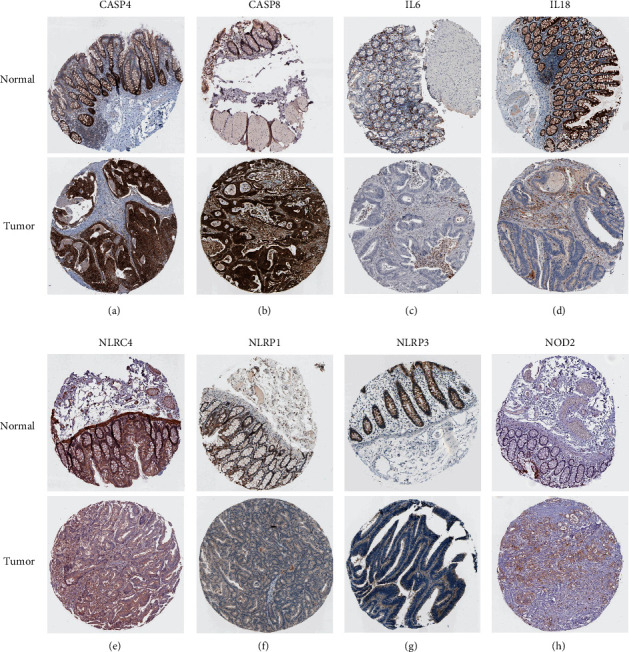
Verification of hub PRG expressions in normal and tumor tissue utilizing the Human Protein Atlas (HPA) database. (a) CASP4; (b) CASP8; (c) IL-6; (d) IL-18; (e) NLRC4; (f) NLRP1; (g) NLRP3; (h) NOD2.

Figure 1 provides an overview of the study flowchart. A total of 39 normal and 398 tumor tissues samples with gene expression were included in the analysis. We found that the majority of the pyroptosis-related genes (23/32, 72%) were significantly differentially expressed between the two groups (*P* < 0.001). 10 genes are upregulated (CASP8, NOD1, GPX4, CASP4, PJVK, IL6, IL1B, PLCG1, NOD2, and GSDMC) and 13 downregulated (ELANE, CASP5, NLRP7, IL18, NLRP3, NLRC4, PRKACA, NLRP1, GSDMB, CASP9, CASP3, TIRAP, and NLRP2) in tumor tissues.  Figure 2(a) shows a heatmap of the expression levels of these genes. To further explore the interactions between the 23 pyroptosis-related DEGs, a PPI network was constructed (Figure 2(b)). The result shows that CASP4, CASP5, and IL18 are at the core of the network. The correlation network containing pyroptosis-related DEGs is presented in Figure 2(c). A total of 11 hub genes including NOD2, CASP4, NOD1, IL18, IL1B, NLRP1, CASP8, NLRC4, IL6, CASP5, and NLRP3 were identified by the MCODE plug-in in Cytoscape software (Figure 2(d)), and their protein levels were verified using the Human Protein Atlas (HPA) database (Figure 3).

### 3.2. Tumor Classification Based on the DEGs

**Figure 4:**
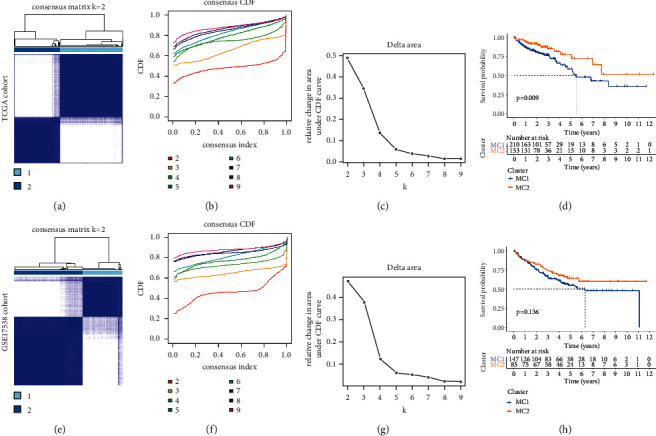
The patient classification based on the pyroptosis-related DEGs. (a and e) Patients in two cohorts were grouped into two clusters according to the consensus clustering matrix (*k* = 2). (b and f) CDF curves of the consensus score (*k* = 2–9) in the two cohorts. (c and g) Relative change in the area under the CDF curve (*k* = 2–9) in the two cohorts. Kaplan–Meier survival analyses of the patients with MC1 and MC2 in the TCGA (d) and GSE17538 (h) cohorts.

**Figure 5:**
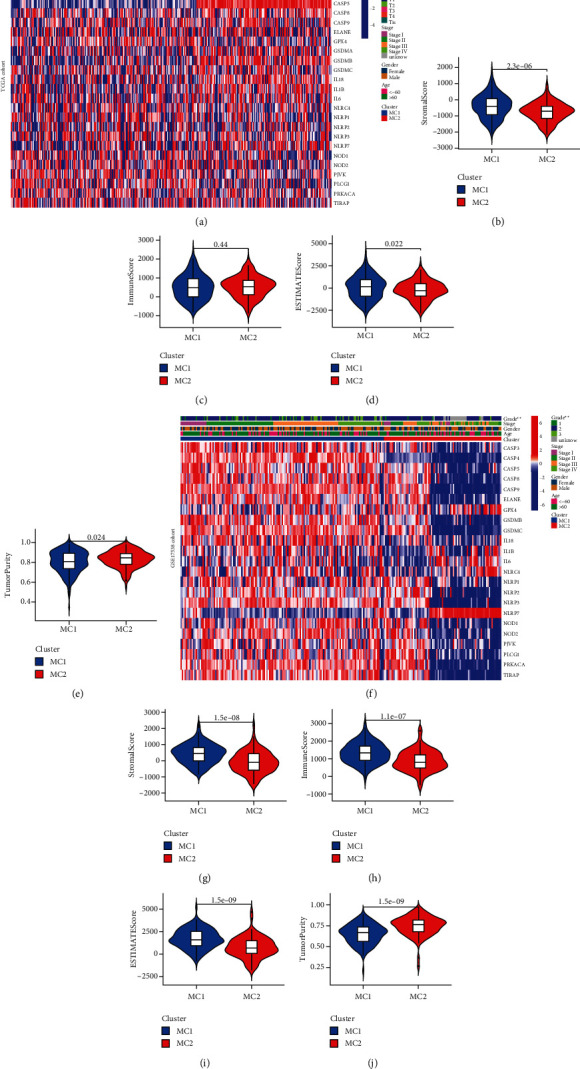
Characteristics of different cluster patients. Heatmap and clinicopathological features of the two MCs in the TCGA (a) and GSE17538 (f) cohorts. Comparison of stromal (b and g), immune (c and h), ESTIMATE (d and i), and tumor purity (e and j) scores in two MCs presented in Violin Plot.

To establish a classification of CC based on the expression patterns of PDGs, machine learning-based unsupervised consensus clustering was performed on CC patients from the TCGA cohort and GSE17538 cohort, respectively (Figures 4(a) and 4(e)). According to the relative change in the area under the cumulative distribution function (CDF) curve and consensus heatmap, the optimal number of clusters was determined to be two (*k* value = 2), and no appreciable increase was observed in the area under the CDF curve (Figures 4(b)-4(c) and Figures 4(f)-4(g)). Thus, all CC patients were classified into two groups: molecular cluster 1 (MC1, 58% in TCGA and 63% in GSE17538) and molecular cluster 2 (MC2 42% in TCGA and 37% in GSE17538). Kaplan–Meier survival analysis indicated that patients with MC1 represented significantly poorer OS than patients with MC2 (Figures 4(d) and 4(h)). The gene expression level and the clinical features between two clusters are presented in a heatmap. We can see that the pathological N stage was significantly different between the two clusters in the TCGA cohort (Figure 5(a)), and the pathological grade was significantly different between the two clusters in the GSE17538 cohort (Figure 5(f)). We further analyzed the difference between MC1 and MC2 on the tumor immune microenvironment. The results revealed that the patients with MC1 in the TCGA cohort had significantly higher immune scores and lower tumor purity than those with MC2 (Figures 5(b)–5(e)). Similar results were observed in the GSE17538 cohort (Figures 5(g)–5(j)).

### 3.3. Development and Validation of a Prognostic Risk Signature

**Figure 6:**
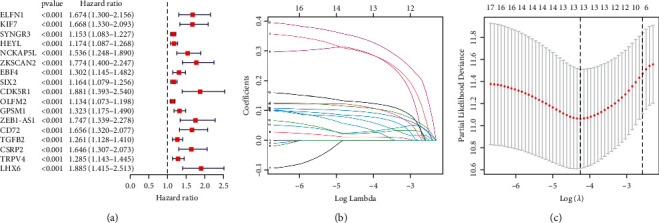
Construction of risk signature in the TCGA cohort. (a) Forest plots showing the results of the univariate Cox regression analysis between DEGs expression and OS. (b) LASSO regression of the 17 OS-related genes (c) Cross‐validation for tuning parameter selection in the lasso regression.

**Figure 7:**
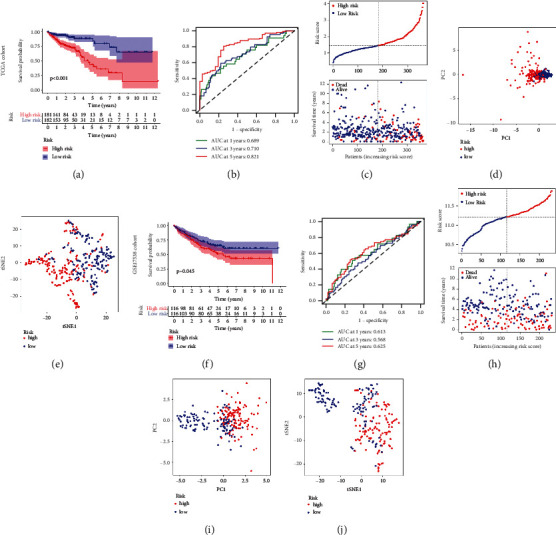
Validation of risk signature in the TCGA cohort and GSE17538 cohort. (a and f) Kaplan–Meier survival curve between high-risk and low-risk groups. (b and g) Plots of the AUC for time-dependent ROC performance (c and h). The distribution and median value of the risk scores and survival statuses of high-risk and low-risk patients. (d and i) Principal component analysis (PCA) plot in the two cohorts. (e and j) t-distributed stochastic neighbor embedding (t-SNE) analysis in the two cohorts.

After obtaining differential genes between MC1 and MC2(*P* < 0.05), we included a total of 363 patients with complete prognosis information for univariate Cox analysis to screen prognosis-related genes from the DEGs by stringent conditions (*P* < 0.0001).  Figure 6(a) presents the result obtained from the preliminary analysis of univariate Cox. In total, there were 17 genes are strongly associated with prognosis. Subsequently, the LASSO algorithm was used to identify the most robust prognostic genes for CC patients (Figures 6(b)-6(c)). A multivariate Cox prediction signature was estimated using the prognostic genes selected by the LASSO. We ultimately identified a 13-gene risk signature. The risk score was calculated as follows: risk score = (0.295 ∗ *KIF7* exp.) + (0.110 ∗ *SYNGR3* exp.) + (0.067 ∗ *NCKAP5L* exp.) + (0.297 ∗ *ZKSCAN2* exp.) + (0.011 ∗ *SIX2* exp.) + (0.096 ∗ *OLFM2* exp.) + (0.028 ∗ *GPSM1* exp.) + (0.340 ∗ *ZEB1-AS1* exp.) + (0.127 ∗ *CD72* exp.) + (0.114 ∗ *TGFB2* exp.) + (0.023 ∗ *CSRP2* exp.) + (0.081 ∗ *TRPV4* exp.) + (0.043 ∗ *LHX6* exp.). Based on the median score calculated by the risk score formula, 363 patients were equally divided into low- and high-risk groups. The principal component analysis (PCA) and t-distributed stochastic neighbor embedding (t-SNE) analysis showed that patients with different risks were well separated into two clusters (Figures 7(d)-7(e)). Patients in the high-risk group had more deaths and a shorter survival time than those in the low-risk group (Figure 7(c)). A significant difference in OS time was detected between the low- and high-risk groups (*P* < 0.001, Figure 7(a)). Time-dependent receiver operating characteristic (ROC) analysis was applied to evaluate the sensitivity and specificity of the prognostic signature, and we found that the area under the ROC curve (AUC) was 0.689 for 1-year, 0.710 for 3-year, and 0.821 for 5-year survival (Figure 7(b)). In parallel, similar results were validated in the GSE17538 cohort (Figures 7(f)–7(j)).

### 3.4. Clinical Value of Risk Signature

**Figure 8:**
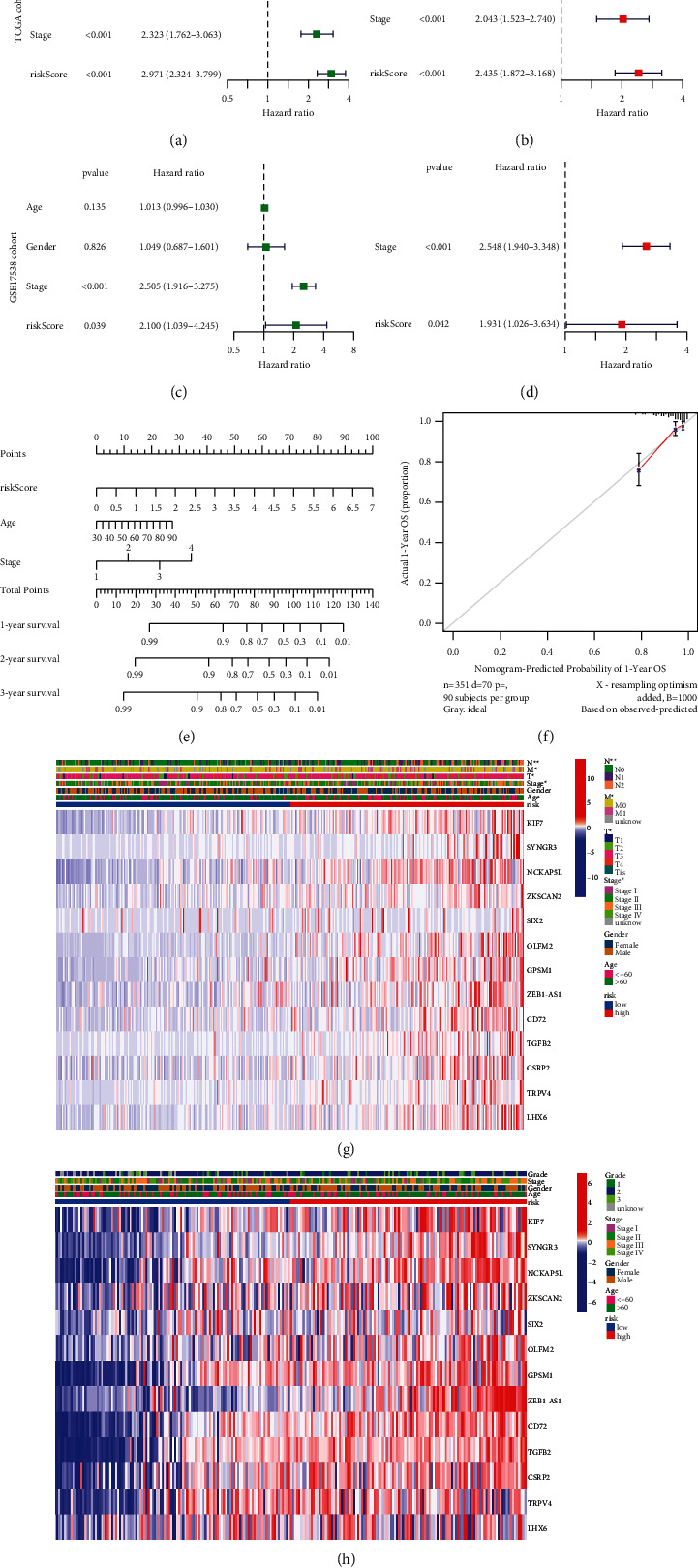
Clinical value of risk signature. (a and c) The univariate Cox regression analysis of the associations between the risk scores and clinical parameters and the overall survival (OS) of patients in two cohorts. (b and d) The multivariate Cox regression analysis of the associations between the risk scores and clinical parameters and the OS of patients in two cohorts. (e) Nomogram to predict the 1-year, 3-year, and 5-year overall survival rates of CC patients. (f) The calibration curve of the nomogram. (g and h) Heatmap for the connections between clinicopathologic features and the risk groups (^*∗*^*P* < 0.05).

To verify the accuracy of the risk signature, we investigated whether the risk score can work as an independent prognostic factor for the survival of CC patients. The risk score and clinical traits such as age, gender, and pathological stage were included in the univariate and multivariate regression analysis. Univariable Cox regression analyses revealed that age, pathological stage, and risk score were significantly correlated with OS (*P* < 0.05, Figure 8(a)). According to the results based on the multivariate analysis, age, pathological stage, and risk score were still confirmed as independent predictors for OS (*P* < 0.05, Figure 8(b)). The independent prognostic value of the risk score was also verified in the GSE15738 cohort (Figures 8(c)-8(d)). On account of the independent predictor factors, a nomogram was established (Figure 8(e)). The calibration curves showed that the nomogram may be an ideal prediction model (Figure 8(f)). The heatmap showed the characteristics of 13-gene expression among different risk groups and their relationship with clinical variables. We can see that, in the TCGA cohort (Figure 8(g)), patients with different risks had significant differences in the clinical characteristics of T stage (*P* < 0.05), N stage(*P* < 0.01), M stage(*P* < 0.05), and AJCC Stage(*P* < 0.05), but the same results were not observed in the GSE17538 cohort (Figure 8(h)).

### 3.5. GO and KEGG Pathway Analysis

**Figure 9:**
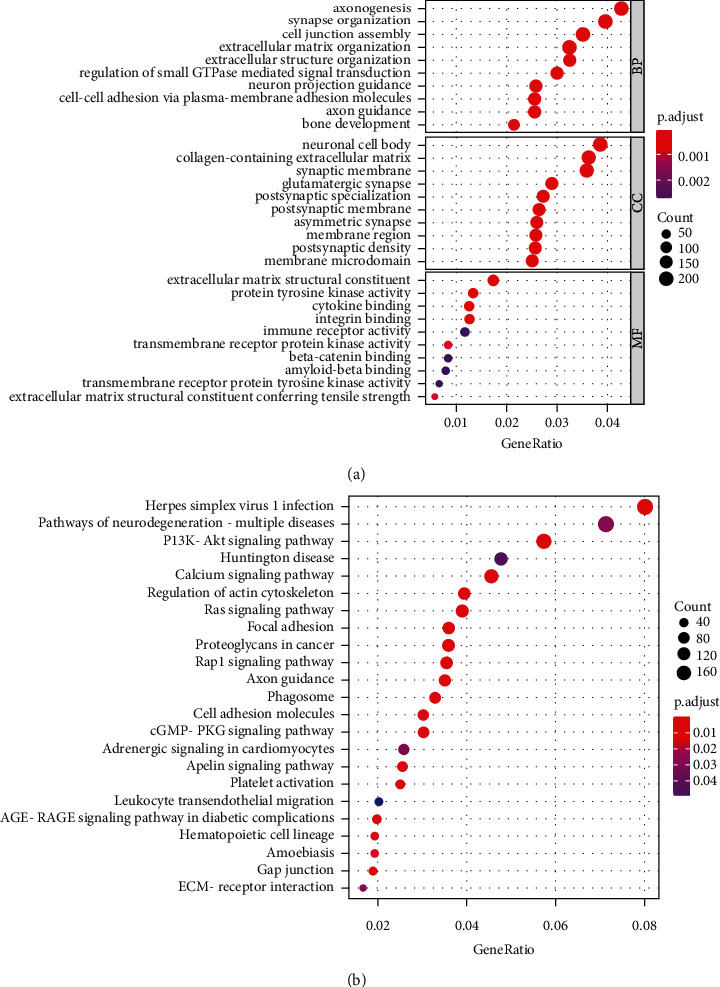
Enrichment analysis of differential genes between high and low-risk groups. (a) Bubble graph for GO enrichment. (b) Bubble graph for KEGG pathways.

To explore the underlying molecular mechanism of the risk signature, we identified differentially expressed genes between the high-risk and low-risk groups (*P* < 0.05). GO and KEGG pathway enrichment analyses were conducted on DEGs. GO enrichment showed that these genes were mainly enriched in cell junction assembly, extracellular matrix organization, extracellular structure organization, collagen-containing extracellular matrix, membrane region, extracellular matrix structural constituent, cytokine binding, and immune receptor activity, etc. (Figure 9(a)). The KEGG pathway showed that the differential gene pathway mainly involves the PI3K-Akt signaling pathway, calcium signaling pathway, Ras signaling pathway, etc. (Figure 9(b)).

### 3.6. Immune Characterizations Analysis and Immune Checkpoint Prediction

**Figure 10:**
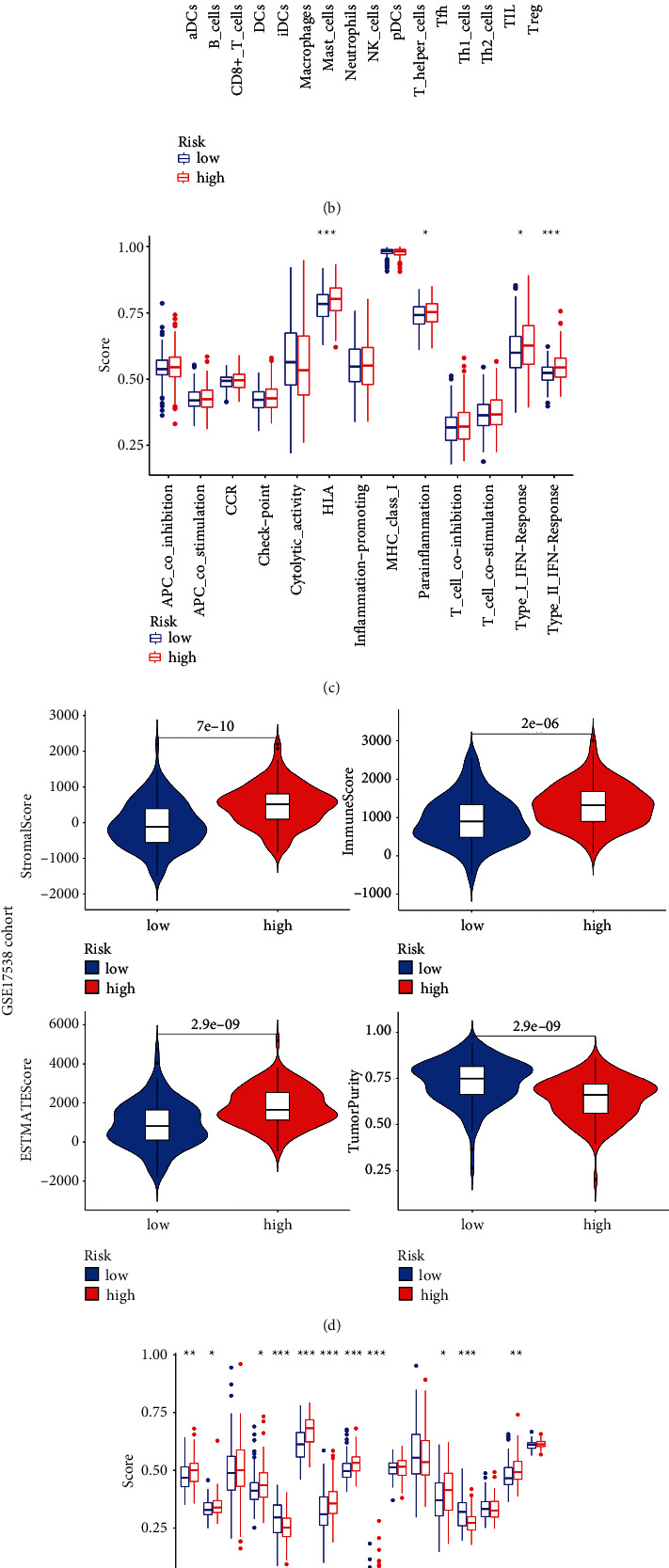
Analysis of immune microenvironment. (a and d) Comparison of stromal, immune, ESTIMATE, and tumor purity scores in high-risk and low-risk groups presented in the Violin Plot. The scores of 16 immune cells (b and e) and 13 immune-related functions (c and f) are displayed in boxplots.

**Figure 11:**
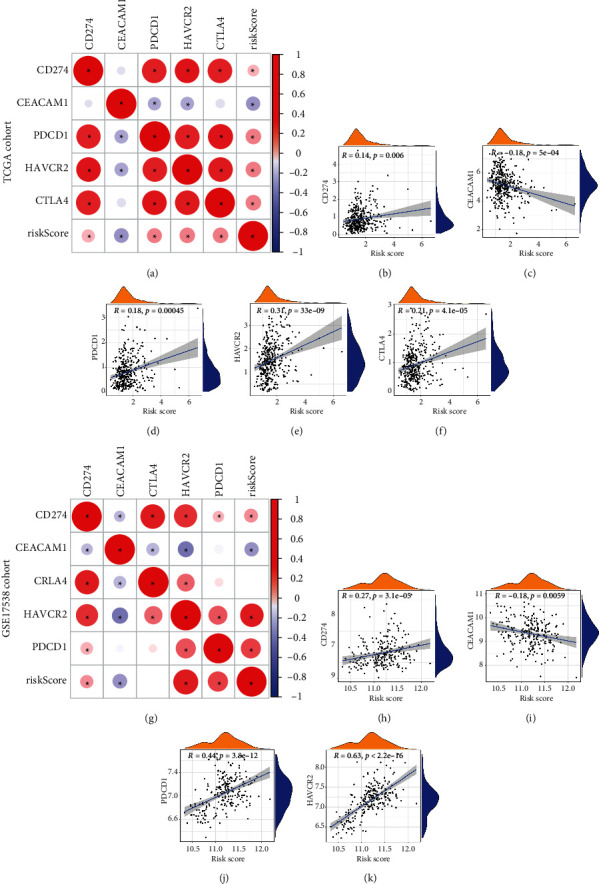
Immune checkpoint analysis. (a and g) Analysis of the correlation between risk score and immune checkpoints. Scatter plot revealing the correlation of risk score and CD274 (b and h), CEACAM1 (c and i), PDCD1 (d and g), HAVCR2 (e and k), and CTLA4 (f).

Through the ESTIMATE algorithm, we explore the different characteristics of the immune microenvironment in the two risk groups, and we further compared the enrichment scores of 16 types of immune cells and the activity of 16 immune-related pathways between the low- and high-risk groups in both the TCGA and GSE17538 cohorts by employing the single-sample gene set enrichment analysis (ssGSEA). The results showed that the stromal score and immune score in the high-risk group were higher than those in the low-risk group. Conversely, the tumor purity of the high‐risk group was unexpectedly lower than the low‐risk group (Figures 10(a) and 10(d)). Specifically, the high-risk group generally had higher levels of infiltration of immune cells, especially active DC cells, B cells, macrophages, neutrophils, NK cells, and TIL cells (Figures 10(b) and 10(e) ). And 4 immune pathways including human leukocyte antigen (HLA), parainflammation, and Type I and Type II IFN response showed higher activity in the high-risk group than those in the low-risk group (Figures 10(c) and 10(f)). Given the importance of checkpoints in immunotherapy, although the checkpoint pathway had no significant difference between the high-risk group and low-risk group, correlation analysis was remained performed between the risk score and checkpoint expression. We found that common checkpoints such as CD274, CEACAM1, HAVCR2, and CTLA4 in the treatment of colon cancer were significantly correlated with risk score (Figures 11(a) and 11(g)). Of these, CD274, PDCD1, and HAVCR2 were positively correlated with the risk score (Figures 11(b), 11(d), 11(e), 11(h), 11(g), and 11(k)), and CEACAM1 was negatively correlated with the risk score (Figures 11(c) and 11(i)). These predictions can help to guide a treatment strategy for targeted drugs.

## 4. Discussion

Although great progress has been made in the treatment and diagnosis of CC compared with the past, the mortality and morbidity of CC are still very high. In particular, CC patients are getting younger and younger, which makes us pay great attention to the early diagnosis and prognosis of CC patients [17]. In the past few decades, many clinical parameters such as age, sex, pathological stage, imaging data, and some serum markers have been widely used to predict the prognosis of patients with CC. However, due to individual differences, the effect of improving treatment and predicting the prognosis of patients through these single factors is often limited. Due to the rapid development of gene sequencing technology, the mRNA levels and mutations of many genes have become predictive markers for the prognosis of malignant tumors. However, because the expression level of a single gene can be regulated by a variety of signal pathways, its predictive effect is usually low. The application of key regulatory factors expression features that play a role in the same signal pathway to construct a polygene model is of great significance to improve the accuracy of prediction and explore new targeted therapies. Some studies in recent years have shown that pyroptosis is involved in the development of CC; targeting pyroptosis was considered as an effective way of overcoming chemoresistance, leading it to be a novel approach for the treatment of CC [12].

In this study, we have collected 32 pyroptosis-related genes reported in public and systematically analyze their expression characteristics in normal and tumor tissues of colon cancer. As expected, 23 (72%) pyroptosis-related DEGs were identified, suggesting that pyroptosis might play an important role in the development of CC. The PPI network constructed by DEGs suggests that NOD2, CASP4, NOD1, IL18, IL1B, NLRP1, CASP8, NLRC4, IL6, CASP5, and NLRP3 are at the core of the network. This is not surprising. Both caspase-4 and caspase-5 are considered to be members of the caspase family associated with inflammation. When a cancer cell is undergoing pyroptosis, a large amount of IL-18 is released out of the cell, reshaping the microenvironment and inducing immune response [18], which may also explain the reason why IL-18 mRNA expression in normal tissue is higher than in tumor tissue. Based on the expression characteristics of these DEGs, we divided the patients into two subgroups (MC1 and MC2). Combined with survival data, we found a trend of worse prognosis in MC1 patients. Heatmap showed that pathological N stage and tumor histological grade were significantly different between the two subgroups in the TCGA cohort and GSE17538 cohort, respectively. When analyzing the tumor microenvironment of the two cohorts, we were surprised to find that their characteristics were consistent; that is, the tumor purity was lower in the MC1 group with a poor prognosis. This finding is consistent with Zhang et al. [19] and Gong et al. [20]. The traditional belief that lower levels of tumor purity often mean stronger immune responses, leading to better prognoses of patients, which is contrary to the findings of the present study. We speculate that there are two possible reasons for this phenomenon. In our previous studies [21], we found that pretreatment systemic inflammatory markers play an important role in the prognosis of patients with locally advanced rectal cancer; among them, the prognostic value of neutrophil-to-lymphocyte (NLR) has a high value in evaluating the survival rate of patients. Hence, it could conceivably be hypothesized that the inflammatory response caused by pyroptosis in the MC1 group leads to changes in systemic inflammatory markers of the whole body, which affects the prognosis of the patients. Another possible reason is cancer metastasis. Heatmap above shows that pathological N stage and tumor histological grade were statistically different between MC1 and MC2. On the basis of this finding, another conclusion can be postulated; that is, the metastasis rate was higher and the tumor differentiation was lower in the MC1 group. Consistent with our finding, Mao et al. [22] also reported that the low tumor purity was related to the poor prognosis of colon cancer.

In order to further explore the value of the two MCs, we constructed a 13-gene signature based on DEGs of MCs (*KIF7*, *SYNGR3*, *NCKAP5L*, *ZKSCAN2*, *SIX2*, *OLFM2*, *GPSM1*, *ZEB1-AS1*, *CD72*, *TGFB2*, *CSRP2*, *TRPV4*, and *LHX6).* Kinesin superfamily (KIF) has a long-reported significant influence on the initiation, development, and progress of cancer. In addition to participating in the Hedgehog signaling pathway [23], *KIF7* was also found to correlate with worse outcomes of breast cancer [24]. Hou et al. [25] and Oliphant et al. [26] both reported the crucial role of SIX homeobox 2 (SIX2) in promoting the stemness of tumor cells, Among them, Michael found that SIX2 can regulate SRY-Box Transcription Factor 2 (SOX2) and induce cancer stem cell programs to mediate advanced metastasis of triple-negative breast cancer, which is in line with our finding that SIX2 was an independent prognostic gene for colon cancer. Moreover, members of the transforming growth factor-*β* (TGFB) family are regarded as the main mediators of epithelial-mesenchymal transition (EMT), which can cause a lack of response to immune checkpoint blockade (ICB). Research results of Yang et al. [27] revealed that TGFB2 could not only play a vital role in linking EMT and tumor mutational burden (TMB) in gastric cancer but also be correlated with poor prognosis. MicroRNA is a class of short noncoding RNA that achieves posttranscriptional regulation by specifically binding to target genes. A variety of miRNAs have been demonstrated to prevent protein expression and affect signaling pathways associated with tumors at the posttranscriptional level and play important roles in multiple stages of colorectal cancer development [28, 29]. LncRNA is another class of ncRNA with a length of more than 200 nucleotides, which are abnormally expressed in various types of tumor cells and are involved in the biological behavior of a variety of malignant tumors. Recent studies have shown that lncRNAs can act as competitive endogenous RNAs (ceRNAs) by binding to response elements (MRE) of microRNAs, thereby indirectly regulating gene silencing resulting from miRNAs [30]. ZEB1-AS1 is a key tumor-associated lncRNA that is considered an oncogenic regulator and a prognostic marker in a variety of malignancies [31, 32]. In colon cancer, on the other hand, several scholars have confirmed that ZEB1-AS1 can bind to multiple miRNAs through the ceRNA mechanism, thereby promoting the proliferation and migration of colon cancer [33–35]. This helps us to demonstrate the accuracy and effectiveness of the model at the experimental level.

Validation of the signature showed a good ability to differentiate patients with different risks. And we found that the risk score calculated by risk signature can work as an independent predictor for OS. Based on the above, a nomogram was established to predict the outcome of CC patients, and calibration curves showed the efficacy of this nomogram. To explore the underlying molecular mechanism of the risk signature, we performed GO and KEGG enrichment analyses. The result showed that DEGs between the high-risk group and low-risk group are involved in both extracellular components and immune receptor activity. In addition, pathways including the PI3K-Akt signaling pathway, calcium signaling pathway, and Ras signaling pathway are enriched. In the same vein, Wu et al. [36] in their research note that the phosphoinositide 3-kinase (PI3K) signaling pathway affects cadherin cell-cell adhesion and also contributes to gastric cancer progression and metastasis. Although the immune-related pathways were less enriched in KEGG results, the increasingly important role of immunotherapy in cancer treatment has to be considered. Hence, the next step of our study was the analysis of immune status in different risk groups. By analyzing the overall feature of the tumor microenvironment, we noticed that the microenvironment characteristics of high- and low-risk groups are in a high level of concordance between those of different MCs patients mentioned above. That is, patients with poorer prognoses had higher immune scores and lower tumor purity in the microenvironment.

We know that inflammation is an important component of the tumor microenvironment and is closely related to the development of tumors. Chronic inflammation-induced genomic mutations are a common premise driving the evolutionary development of colorectal cancer, while cellular mutations promoted by the immune response with dysregulated genomic damage repair are the basic impetus for colorectal cancer cell evolution [37, 38]. Also in the chronic inflammatory microenvironment, mutant cells that survive selection and survival competition often cause changes in the conduction pattern of key signaling pathways in the cell; for example, there is persistent activation of the Wnt signaling pathway in links that promote intestinal adenoma production, which can break the regulatory balance of normal intestinal epithelial cell proliferation, differentiation, and apoptosis [39]. In addition, primitive mutant cells often get the ability of stem cell activity and immune tolerance. Therefore, different microenvironments may affect the risk of cancer in patients with different intestinal diseases such as inflammatory bowel disease, microcolitis, and irritable bowel syndrome [40, 41]. Our results demonstrated the above theory that patients in the high-risk group have a tumor immune infiltrating microenvironment with a worse prognosis.

In addition, our study also found that the characteristics of tumor purity in different risk groups were highly consistent with the above MC classification. This also flanks that our prognostic model is robust. However, whether tumor purity represents biological relevance and intrinsic characteristics of the tumor, or just systemic bias determined by external characteristics, such as surgical resection and tissue preparation, is still controversial [42]. Thus, tumor purity has important clinical, genomic, and biological significance and is an important confounding factor in cancer genome or transcriptome analysis. We need to make a specific analysis of different specific tumors. In our study, we found that the expressions of active DC cells, B cells, macrophages, neutrophils, NK cells, and TIL cells were higher in the high-risk group than those in the low-risk group in both TCGA and GSE17538 cohort, indicating stronger immune functions in the high-risk group. Immune cells participate in all stages of tumorigenesis and development, showing both antitumor and tumor-promoting effects. In the previously mentioned heatmap, we found that the poor prognosis of patients with MC1 type was associated with a low grade of the tumor, which suggests that cancer stem cells (CSCs) may play an important role in the pyrosis-related immune response. A number of emerging experimental evidence have recently confirmed the effects of CSCs on immune cells in the tumor microenvironment, including tumor-associated macrophages and T cells, and the importance of these immune cells in maintaining CSC stemness [43]. CSCs have the ability to evade cell death and metastasize, although they may stay dormant for long periods of time [44]. Considering that a small proportion of tumor cells undergo pyroptosis is sufficient to effectively regulate the tumor immune microenvironment, which in turn activates the T-cell-mediated antitumor immune response [45], and T cells have an activating effect on cancer stem cells [46], a possible explanation of our study result is that pyroptosis remodels the immune microenvironment, which activates the ability of cancer stem cells to metastasize to distant sites, leading to poor patient prognosis. Thus, the effects of immune checkpoints in high-risk patients deserve further exploration. We selected immune checkpoints common in colon cancer therapy for analysis, finding that CD274, PDCD1, and HAVCR2 were positively correlated with the risk score. Among them, HAVCR2, which coding TIM-3, showed the highest association with the risk score (*R* = 0.31 or 0.63 in TCGA and GSE17538, respectively). So far, a large number of studies [47] have confirmed that Tim-3 plays an inhibitory role in antitumor innate immunity, and the targeting drugs based on TIM-3 are also in clinical trials [48], which suggest that we can improve the prognosis of patients in the high-risk group by targeting TIM-3.

The purpose of the current study was to determine expression features and prognostic value of pyroptosis-related genes in CC. The results of this study show that most of the pyrogenic genes have significant expression differences between tumor and normal tissues. Based on the expression characteristics of DEGs, we clustered patients and constructed a prognostic signature, and the accuracy of the signature was validated on TCGA and GSE17538 cohorts, respectively. Further analysis suggests that TIM-3 may be a potential target for improving the prognosis of patients in the high-risk group. At present, there are few studies on pyroptosis in colon cancer. As far as we know, we first systematically reported the prognostic value of pyroptosis-related genes in colon cancer; these findings have significant implications for the understanding of the role of pyroptosis-induced changes in the immune microenvironment in the development of colon cancer, which provides a theoretical basis for future research. However, the present study has several limitations that need to be acknowledged. First, this signature only employs retrospective data from two databases, and further prospective studies are needed to prove its clinical value. Secondly, the role of pyroptosis in the tumor immune microenvironment and the therapeutic effect of targeted TIM-3 on patients require more verification by in vitro experiments and clinical trials. Thirdly, colonoscopy is so far considered the gold standard for diagnosing colorectal pathology [49]. Aggressive colonoscopy can improve the early detection rate of colorectal cancer. However, as an invasive examination, the complications caused by colonoscopy, such as perforation and bleeding after polypectomy, are inevitable. In addition, it has been reported that a lesion miss rate ranging between 6% and 12% for large polyps and 5% for cancers has been described [50]. Therefore, it is a better choice to select an experienced endoscopist for examination. However, there are no reports that targeted colonoscopy screening and monitoring policies will curb the rise in colorectal cancer incidence, and the scope of colonoscopy combined with biological sample analysis is also limited to feces, blood, and urine [51]. Therefore, this study may be more suitable for patients with confirmed colon cancer, and more targeted treatment can be performed through the screening of patients with different prognostic risks.

## 5. Conclusion

In conclusion, our study provides a good prognostic signature for patients with colon cancer, and the validity and reliability of this signature are verified in two datasets. Importantly, we provide a prognostic nomogram to participate in clinical treatment decisions and provide potential targets for targeted therapy in patients, providing us with new insights into the development of colon cancer and discovering new therapeutic methods.

## Data Availability

The raw transcriptome data of this study are available in the TCGA database (TCGA-COAD cohorts) (https://portal.gdc.cancer.gov/) and the GEO database (GSE17538), which are publicly available databases.
